# Lying-In Hospital, Dublin

**Published:** 1831-08

**Authors:** 


### LYING-IN HOSPITAL, DUBLIN.

### *Two Cases of Recovery from Laceration of the Uterus and Vagina.* By Robert Collins, m.d.*

Case I. Jemima Day, aged twenty-five, was admitted into the hospital in labour of her third child (a boy), with the hand and arm protruded out of the vagina as far as the elbow. The funis was prolapsed, and without pulsation.

She had been attended, previous to admission, by two surgeons and a midwife, and it was reported the midwife had mistaken the hand for the foot, and pulled it down.

When admitted, she was in a very debilitated state, with a feeble quick pulse, ghastly countenance, expressive of much anxiety. It was evident she had suffered some most serious injury, and, from the symptoms present, rupture of the vagina or uterus was too apparent. She had had sixty drops of tincture of opium before admission.

On examination per vaginam, the shoulder and body of the child were found to be forced so low, and so firmly fixed in the pelvis, as to completely forbid any attempt to turn the child; nor was there any necessity to do so, as it was dead. The thorax of the child was then perforated and broken down, and the breech was afterwards brought down with the crotchet, without the least difficulty.

On introducing the hand into the vagina, after the child and placenta were taken away, an extensive laceration was found at the junction of the cervix uteri with the vagina posteriorly.

I placed the parts as nearly as possible in their natural position, guarding carefully against any portion of the intestines being included in the lacerated part; at the same time removing as much of the clotted blood as could be got away.

It was evening when she was admitted into the hospital; and after the delivery, &c. was completed, and her bed made dry, she was ordered a powder containing ten grains of calomel and the same quantity of jalap, which remained on the stomach, and operated freely on the bowels.

The following morning, the pulse was 130; there was much tenderness of the abdomen, and she had rested little. Three dozen leeches were ordered to the abdomen, and a warm bath afterwards; she was directed also to be stuped every third hour, with flannels wrung out of boiling water, as hot as could be endured.

Much relief was procured by these means, yet considerable tenderness still remained: however, by repeating the leeches and warm bath, with stuping every third hour, the distress subsided in a great measure before the end of the fourth day. The pulse gradually returned to its natural state; she became stronger every day, and she left the hospital perfectly well on the twenty-third day after delivery, and returned at the end of three months in good health.

* Dublin Med. Transactions, vol. i. part i. New Series.

There was no other treatment adopted in this case but what is stated above, except that the strictest attention was paid to the regulation of her bowels; and the medicines used for this purpose were castor oil, infusion of senna, with sulphate of magnesia and tincture of jalap, or the common saline effervescing draught, made with the carbonate of soda, with the addition of one ounce of tartrate of soda and potash to eight ounces, given with lemon juice. Her diet was also attended to, and nothing either difficult of digestion or stimulating was given. Fruit of different kinds, both raw and stewed, whey, gruel, tea, flummery, and latterly broths, were her chief food. The bowels, from the commencement, were easily affected by the smallest quantity of medicine.

Case II. Anne Woodward, aged thirty, was admitted in labour of her sixth child (a girl), at six o'clock in the evening of the 27th of October. Her labour was reported to have commenced seven hours previous to admission.

When she came into the hospital, the uterus was acting briskly, and the head of the child advanced rapidly, so much so that those who were in attendance thought it would have been expelled every pain: suddenly, however, the uterine action completely ceased, and considerable debility, great distress of countenance, vomiting, and other symptoms strongly indicating rupture of the uterus, ensued.

The head of the child was low down, and pressing on the neck of the bladder, so much so that the catheter could not be introduced; and, as immediate delivery was necessary, the head was lessened, and the child brought away with the crotchet. The uterus assisted strongly in expelling the child and placenta: however, on introducing the hand into the vagina afterwards, a most extensive laceration was found at the junction of the cervix uteri with the vagina anteriorly, and the intestines had fallen through the opening into the vagina.

After returning the intestines carefully, the edges of the laceration were brought as nearly as possible in contact, and the patient was enjoined to remain perfectly quiet during two hours on the couch, where she was delivered. She was then cautiously carried to bed, and a powder containing eight grains of calomel and fifteen of jalap, with half a grain of powdered opium, administered.

Following morning, October 29th, nine o'clock. Pulse 114 and feeble; rested badly; abdomen distended, and much tenderness on pressure; bowels have not been opened.—To have one ounce of castor oil and one of tincture of jalap immediately; three dozen leeches to the abdomen, and afterwards to be put in the warm bath, and permitted to remain in it as long as she finds it agreeable; the abdomen to be fomented every second hour, with flannels wrung out of boiling water, as hot as the patient can bear. The oil and tincture of jalap to be repeated in three hours, if the first had no effect.

Nine o'clock p.m. Pulse 120; tongue foul; purgative draught was repeated; bowels have been well emptied; abdomen softer. and less painful on pressure. Stuping to be diligently continued.

30th, nine o'clock A.M. Pulse 114; tongue foul; had some rest; abdomen full; uterus enlarged, and much tenderness on pressure; bowels open.—Three dozen leeches to the abdomen, in the region of the uterus; afterwards a warm bath; stupes to be continued every second hour. To take the common saline effervescing draught, with the addition of one ounce of tartrate of soda and potash to eight ounces.

Nine o'clock P.M. Pulse 120; felt relief from the leeches and bath; bowels open; abdomen softer, and much less painful on pressure.—Fomentations and saline draughts to be continued.

31st, nine A.M. Pulse 114; rested tolerably; drank freely; bowels open: uterus still continues enlarged, hard, and tender on pressure.—Three dozen leeches and warm bath to be repeated; stuping to be continued every second hour, and to have three drachms of castor oil in an ounce of pennyroyal water.

November 1st, nine A.M. Pulse 114; rested well; drank freely; abdomen soft and free from pain; uterus still hard and enlarged; bowels open.—Fomentations to be continued to the abdomen, and the saline effervescing draught, wiih Rochelle salt, to be repeated.

2d, nine A.M. Pulse 114; tongue moister and cleaner; rested well; drinks freely, and feels easy, except when she moves in the bed; abdomen nearly free from pain; bowels open.—Stupes to be continued occasionally.

3d, nine A.M. Pulse 114: in every respect improved since last visit. She gradually continued to amend, and was discharged perfectly well on the 30th of November, one month and two days from the date of her delivery.

Her pulse, from the time of delivery, was feeble, and continued, for the first twelve days, regularly to beat 114 in the morning, and 120 in the evening: at the end of this time it fell to ninety-eight, and gradually became stronger and more natural. On the fifteenth and sixteenth days after delivery, there was a very considerable discharge of unhealthy pus from the vagina, to the amount perhaps of a pint in the first instance, and less the second: it had probably collected about the lacerated part. However, it did not interfere with her recovery, and her strength being supported by nutritious diet, she was not reduced by it. After the first twelve days, she was liberally supplied with chicken broth, chicken, stewed apples, grapes occasionally, and a little wine; she also got the cold infusion of bark, in the form of an effervescing draught. She had no medicine of any kind after the first four days, except the bark draught, and occasionally the saline effervescing draught, with Rochelle salt, or three or four drachms of castor oil, to keep the bowels gently open.

The bowels from the commencement in both cases, the reader may observe, were easily acted on by the smallest doses of medicine, after having, in the first instance, been well emptied; which greatly contributed to the favorable termination of both.

